# Differentiating Ischemic From Nonischemic T-Wave Inversion Using a Multimodal Vision-Language Model With Reinforcement Learning (ECG-R1): Development and Validation Study

**DOI:** 10.2196/87227

**Published:** 2026-06-19

**Authors:** Yunzhang Cheng, Zhongkai Wang, Wen Zhang, Qin Zhang, Mingwei Zhang, Songbin Cai, Tianyi Zhang

**Affiliations:** 1 School of Health Science and Engineering, University of Shanghai for Science and Technology, Room 1002, 10th Floor, Zhuoyue Building, No. 334 Jungong Road, Shanghai, Yangpu District, 200093, China, +86 18566664556; 2 Department of Cardiovasology, Changhai Hospital, Shanghai, Shanghai, China; 3 Xin Hua Hospital Affiliated to Shanghai Jiao Tong University School of Medicine, Shanghai, China

**Keywords:** electrocardiogram, ECG, T-wave inversion, myocardial ischemia, computer-aided diagnosis, vision-language models, multimodal artificial intelligence, reinforcement learning, artificial intelligence, AI

## Abstract

**Background:**

The differentiation of primary ischemic from secondary nonischemic T-wave inversion (TWI) on electrocardiograms (ECGs) presents a critical and pervasive diagnostic challenge in emergency cardiology. Historical clinical literature reports that clinician-led visual interpretation of isolated TWI yields a positive predictive value of only approximately 50% due to profound morphological ambiguity. This high degree of uncertainty frequently leads to high false-positive rates, resulting in unnecessary, costly, and potentially risky invasive angiographic procedures for patients. Furthermore, although existing deep learning models have attempted to address this clinical bottleneck, they are frequently limited to single-modality, “black box” architectures. Their inability to process complex multimodal data or provide transparent reasoning traces fundamentally limits clinical trust and real-world adoption.

**Objective:**

The objective of this study was to develop a novel diagnostic framework designed to address the critical clinical challenge of accurately differentiating ischemic from nonischemic TWI. By using a multimodal vision-language model trained with a reinforcement learning (RL) paradigm, this study aimed to improve diagnostic accuracy and provide interpretable reasoning.

**Methods:**

We developed ECG-R1, a multimodal framework using the Qwen2-VL-2B vision-language model, to analyze ECG waveform images and associated clinical text. Instead of supervised fine-tuning (SFT), the model was trained using an RL paradigm with the group relative policy optimization algorithm. The model was trained to generate a structured output containing an explicit reasoning trace and a final “yes” or “no” answer. A 2-component, rule-based reward function was designed to assess format adherence and diagnostic accuracy. Performance was compared against strong SFT baselines.

**Results:**

Evaluated on a large-scale multimodal dataset of 12,917 TWI cases, our ECG-R1 model achieved a state-of-the-art in-domain accuracy of 75.21%, a sensitivity of 82.55%, and an area under the receiver operating characteristic curve of 84.18%. The model demonstrated robust cross-hospital generalization, maintaining a 72.93% out-of-domain accuracy and an 81.56% area under the receiver operating characteristic curve. When controlling for model scale, the RL paradigm yielded substantial absolute improvements of 6.69% in in-domain performance and a substantial 11.48% improvement in out-of-domain performance over the capacity-matched Qwen2-VL-2B full-FT baseline. These results suggested that the RL approach was superior for learning invariant physiological features rather than overfitting to source-domain artifacts.

**Conclusions:**

The RL-based ECG-R1 framework significantly outperformed capacity-matched SFT baselines in both diagnostic accuracy and cross-domain robustness. By explicitly modeling interpretable clinical reasoning and using probabilistic diagnostic language to prevent premature cognitive closure, ECG-R1 may serve as a highly transparent clinical decision support system. It was structurally designed to safely assist cardiologists within a strict human-in-the-loop paradigm, establishing a robust foundation for prospective clinical trials.

## Introduction

Cardiovascular diseases remain the principal cause of mortality worldwide [1]. Myocardial ischemia, defined as an inadequate supply of blood to the heart muscle, represents a critical pathophysiological precursor to serious cardiac events, including myocardial infarction [2]. The electrocardiogram (ECG) is the primary frontline tool for diagnosing ischemia due to its noninvasive nature, low cost, and accessibility [3-5]. However, a significant challenge lies in interpreting common but ambiguous ECG findings, such as T-wave inversion (TWI) [6]. While TWI can indicate ischemia, it is a notoriously nonspecific marker of an exceptionally broad range of causes, from benign physiological variations to life-threatening conditions such as acute coronary syndrome [7-9]. This diagnostic ambiguity creates a clinical dilemma, as clinical studies and standardized ECG guidelines indicate that the positive predictive value of isolated TWI for true myocardial ischemia is limited to approximately 50%, largely due to profound morphological confounding from nonischemic etiologies [10,11]. The resulting high false-positive rate burdens health care systems with unnecessary, costly, and risky investigations, such as invasive coronary angiograms, and causes significant patient anxiety [12-14]. Consequently, developing a reliable methodology to accurately and efficiently differentiate between truly ischemic and nonischemic TWI from this vast patient population remains a critical and pressing unmet need in modern cardiology [15-17].

In recent years, deep learning has revolutionized automated ECG analysis. Early supervised fine-tuning (SFT) approaches used 1D convolutional neural networks to extract discriminative features from raw signals. Subsequent SFT-based models integrated recurrent neural networks to capture temporal dependencies, while recent transformer architectures leverage self-attention under an SFT framework to model long-range contexts, achieving state-of-the-art performance in arrhythmia detection [18-20]. Despite their increasing sophistication, these methods are constrained by several fundamental limitations that hinder their clinical applicability. First, most existing models operate in a single-modality vacuum, analyzing only the raw ECG signal data while completely disregarding the rich, contextual information embedded in the accompanying clinical text, such as the preliminary impressions of the reporting cardiologist or the documented history of the patient [21]. Second, these models largely function as inscrutable “black boxes.” They provide a diagnostic output without offering any insight into their decision-making process, a critical deficiency in the high-stakes medical domain where transparency, trust, and clinical validation are paramount [22-24]. Finally, the prevailing training paradigm of SFT has inherent weaknesses. SFT often encourages models to engage in “shortcut learning”—relying on spurious correlations in the data rather than true pathological patterns [25,26]. This leads to a form of rote memorization that results in poor generalization to new, unseen data, a limitation aptly captured by the recent observation that “SFT Memorizes, RL Generalizes” [27,28].

To address these challenges, we propose ECG-R1, a novel intelligent diagnostic framework built upon a multimodal vision-language model (VLM). By leveraging a 2-billion parameter model, our framework is designed not only for high performance but also for practical clinical deployment, balancing advanced capabilities with computational efficiency. This approach pioneers the integration of visual ECG waveform data with the semantic content of textual reports. This synergy enables a more holistic and contextualized analysis for understanding the clinical state of a patient. Critically, we move beyond the traditional SFT paradigm by using a sophisticated reinforcement learning (RL) framework, specifically applying the group relative policy optimization (GRPO) algorithm [29]. Unlike SFT, which often leads to rote memorization and poor generalization, our RL approach incentivizes the model to autonomously explore data, identify robust pathological features, and generate transparent, human-understandable reasoning pathways. This RL mechanism directly addresses the critical “black box” problem prevalent in medical artificial intelligence (AI) by making the step-by-step decision logic of the model explicit and independently verifiable by clinicians. Ultimately, our method culminates in a powerful multimodal diagnostic tool. Evaluated on a strictly held-out test set, ECG-R1 not only achieves an in-domain accuracy of 75.21% in distinguishing true-positive from false-positive ischemic cases but also demonstrates a high sensitivity of 82.55% and an impressive area under the receiver operating characteristic curve (AUC-ROC) of 84.18%. Furthermore, the model exhibits robust cross-hospital generalization, maintaining a 72.93% accuracy and an 81.56% AUC-ROC in out-of-domain evaluations. By generating a transparent reasoning trace alongside these highly reliable, probabilistic diagnostic assessments, ECG-R1 provides a trustworthy evidence base to safely support clinical decision-making in high-pressure emergency environments.

## Methods

### Overview

We used RL to enhance explicit reasoning capabilities in medical VLMs, adopting GRPO for its efficiency and demonstrated effectiveness. While GRPO has been primarily applied to reasoning tasks in coding and mathematics, we adapted this framework to the medical domain, with a focus on ECG analysis. Our approach integrated ECG waveform images as visual inputs and incorporated custom reward functions that promote structured clinical reasoning and domain-compliant response formats.

### Ethical Considerations

This retrospective study used strictly deidentified clinical data, with all protected health information fully redacted from ECG images and textual reports to ensure patient privacy and safety. Because this research involved exclusively the secondary analysis of pre-existing, fully anonymized data, it was exempt from formal ethical review and the requirement for an approval number by the institutional ethics committees of the First Affiliated Hospital of Naval Medical University (Changhai Hospital) and Shanghai Xinhua Hospital (Xin Hua Hospital). This exemption was in accordance with Article 32 of the “Ethical Review Methods for Life Sciences and Medical Research Involving Humans” (2023) issued by the National Health Commission of the People’s Republic of China [30]. For prospective clinical application, ECG-R1 was designed strictly as an assistive clinical decision support system operating under a “human-in-the-loop” paradigm.

### Data Acquisition and Preprocessing Pipeline

The dataset for this study was sourced from the First Affiliated Hospital of Naval Medical University (hospital A), comprising 12,917 cases, and Shanghai Xinhua Hospital (hospital B), comprising 1356 cases. Each case included both an ECG waveform image and associated text. Representative, deidentified examples illustrating the varying quality and format of this textual modality—including cardiologist impressions and patient histories—are provided in Table S1 in Multimedia Appendix 1. A key characteristic of this dataset was that all ECG images exhibited TWI. The dataset was well-distributed across patients of varying ages, genders, and medical histories. The ground truth for myocardial ischemia was established based on coronary angiography (CAG), the clinical gold standard. In cases where multiple CAG records were available, the one closest to the ECG acquisition time was used. This ensured that the model learned to identify waveform features associated with proven anatomical obstruction rather than subjective clinician interpretation. Data from hospital A (in-domain) were randomly partitioned at the patient level into training and internal test sets at a 7:3 ratio. In contrast, the entire dataset from hospital B served as an out-of-domain external validation set to assess model generalizability.

**Figure 1.**
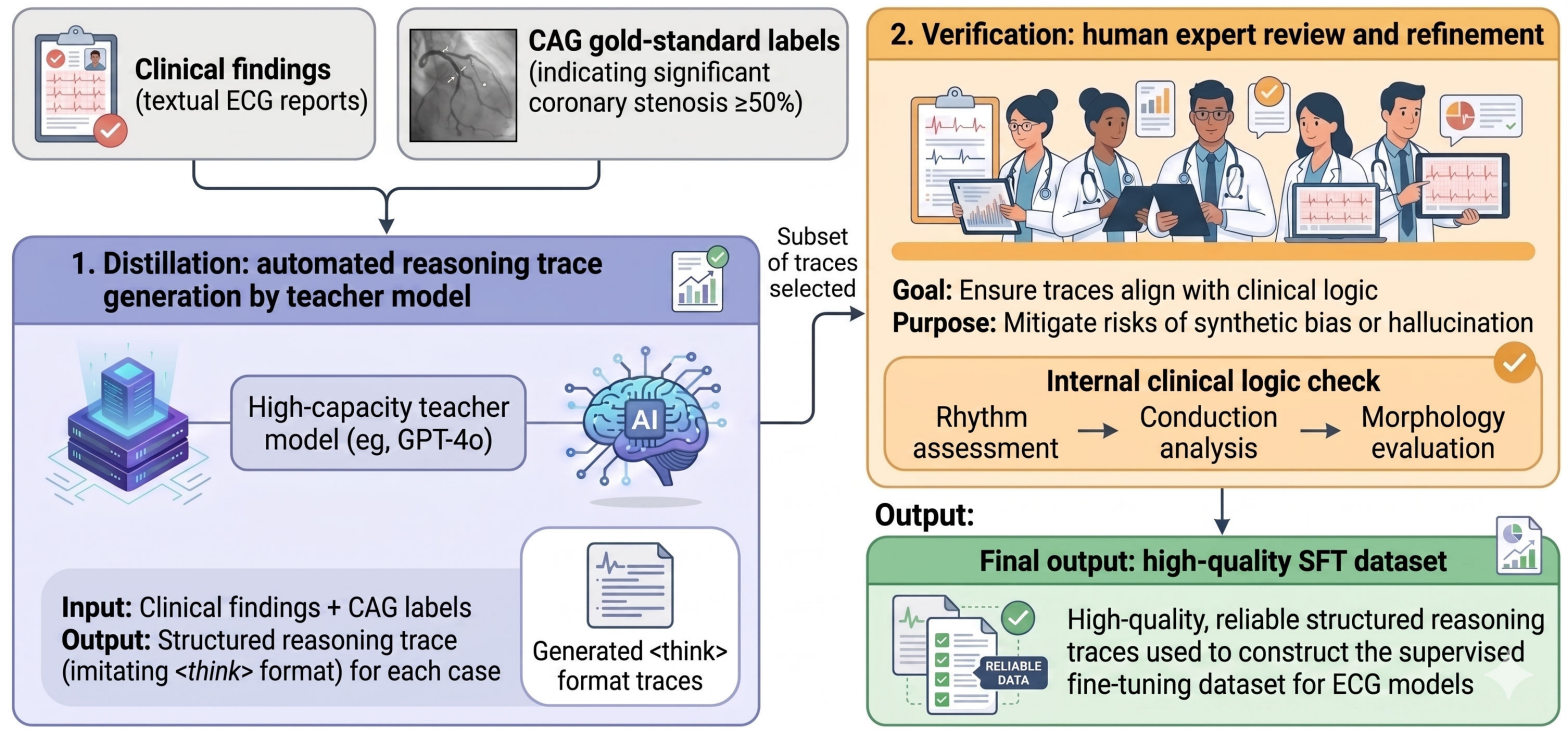
Schematic illustration of the “Distillation-then-Verification” pipeline for supervised fine-tuning (SFT) dataset construction. CAG: coronary angiography; ECG: electrocardiogram.

To construct a high-quality SFT dataset, we implemented a “Distillation-then-Verification” pipeline (Figure 1). First, a teacher model (GPT-4o; OpenAI) generated structured reasoning traces (<think> format) based on clinical findings and CAG gold-standard labels (≥50% stenosis). Subsequently, to mitigate synthetic hallucinations, expert clinicians verified the traces against strict clinical logic—covering rhythm, conduction, and morphology—thereby ensuring the reliability of the final dataset.

**Figure 2.**
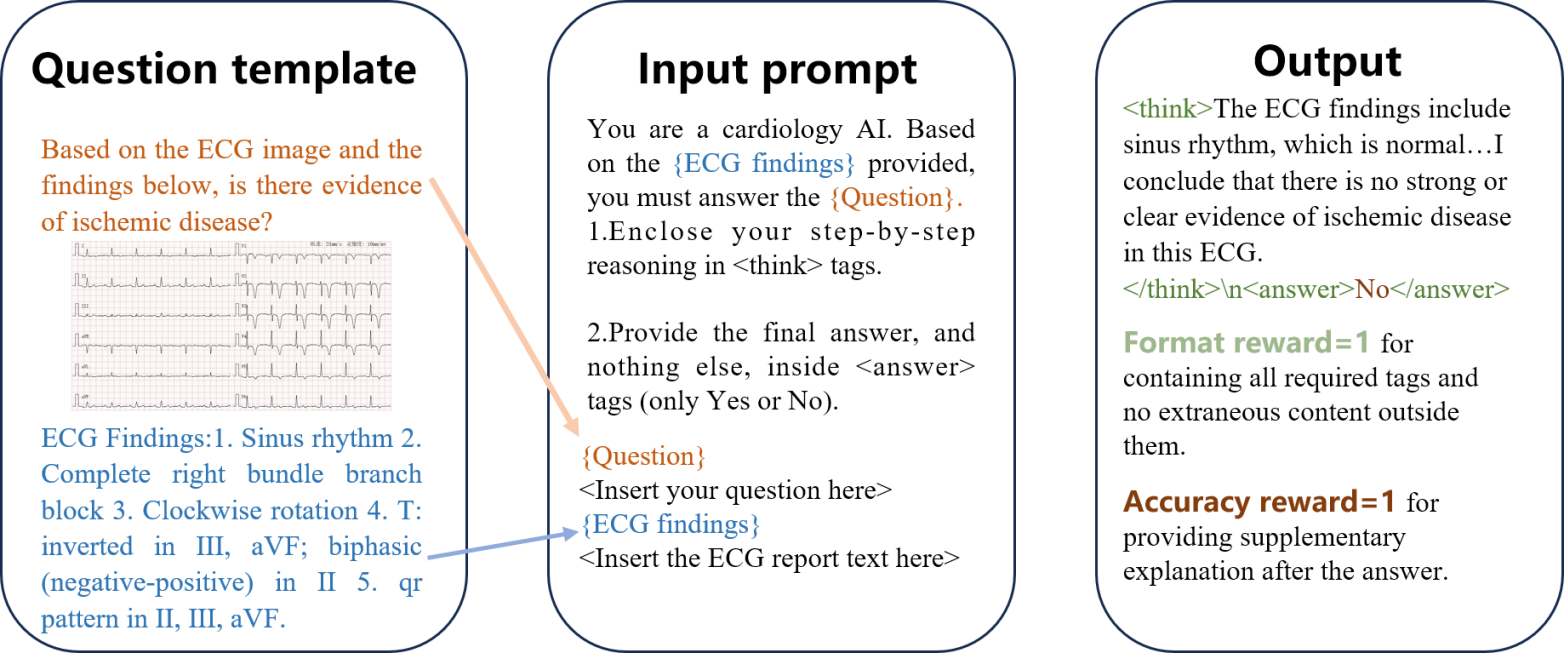
The process illustrates how multimodal inputs (electrocardiogram [ECG] image and text report) are integrated into a structured prompt, guiding the model to generate an output containing its reasoning (<think> tag) and final answer (<answer> tag). The system then assigns rewards based on format and accuracy to optimize the diagnostic capabilities of the model.

We used the Qwen2-VL-2B as our base model, denoted by $\pi_{\theta }$, where θ represents its trainable parameters. The model was trained on a dataset, where each sample consisted of (1) an image depicting an ECG waveform and (2) a text prompt comprising a user question and a fixed system prompt, as shown in Figure 2. The VLM generated an output that includes both a reasoning trace and a final answer, structured within specific XML-style tags (ie, <think>...</think> and <answer>...</answer>). Our RL objective was to optimize $\pi_{\theta }$ such that the model produced accurate answers, adhered to the desired output format, and demonstrated transparent clinical reasoning.

### GRPO Training Pipeline

**Figure 3.**
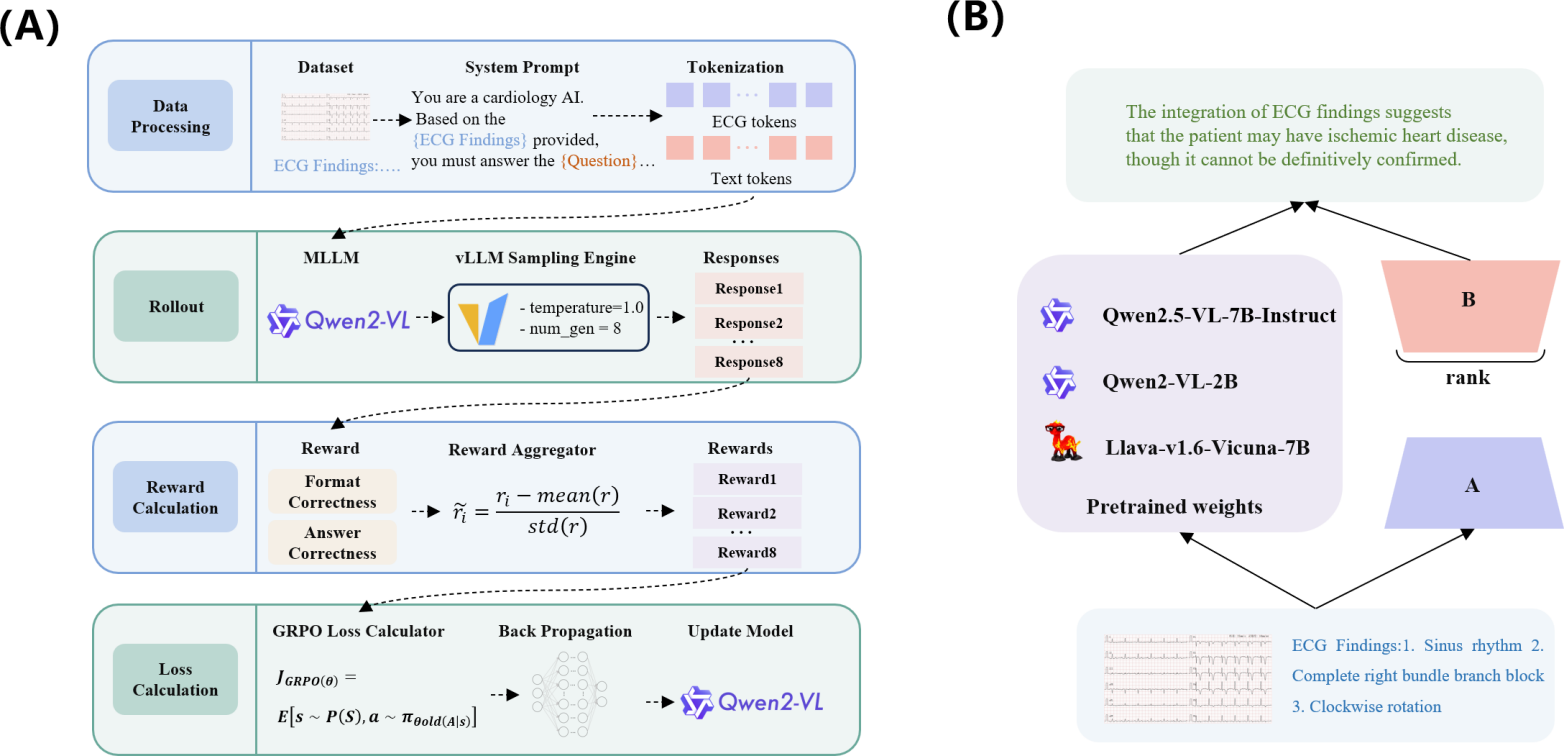
(A) Architectural overview of the group relative policy optimization (GRPO) training pipeline for the ECG-R1 model. The workflow includes data processing, sampling multiple candidate responses from the Qwen2-VL model, calculating rewards based on format and accuracy, and updating the model parameters using the GRPO loss. (B) Illustration of the comparative framework used to evaluate different pretrained vision-language model baselines. AI: artificial intelligence; ECG: electrocardiogram; MLLM: multimodal large language model.

As illustrated in Figure 3A, our model training pipeline used the GRPO algorithm. As a RL algorithm, GRPO directed the policy updates of the model using a reward signal meticulously designed for the task of ECG analysis.

Unlike standard proximal policy optimization, which doubles the memory footprint by requiring a separate value network, GRPO was highly efficient for large VLMs [31]. It extends proximal policy optimization [32] by using a group-relative advantage instead of a learned value function. In each training step, the algorithm began by sampling a group of G candidate outputs ${\left\{O_{i}\right\}}_{i=1}^{G}$ from the current policy, $\pi_{\theta_{old}}$ using vLLM sampling. A core component of this process was the calculation of a reward score $r_{i}$, for each candidate. The effectiveness of this reward signal guided the direction and efficiency of optimization. For our binary ECG classification task (eg, “Is there evidence of ischemic disease?”), we designed a 2-component, rule-based reward function.

First, a format reward incentivized adherence to a predefined output structure. A score of 1 point was awarded if the output strictly contained a single <think> block and a single <answer> block with no extraneous content; any deviation from this format (eg, missing or duplicated tags) yielded 0 points. Conditioned on meeting the format requirements, an accuracy reward was then assessed. If the content within the <answer> tag was an exact, case-insensitive match to the ground-truth label (“yes” or “no”), an additional 1 point was awarded. Any incorrect or verbose answer received 0 points. The total reward for each candidate, $r_{i}$, was the sum of these 2 components, resulting in a value in the range [0,2]. This hierarchical reward structure, which prioritized format before accuracy, guided the model to first master the response schema before optimizing the clinical accuracy of its judgments.

After obtaining the reward $r_{i}$ for each candidate, we calculated the group-relative advantage, $A_{i}=\frac{r_{i}-\mu_{r}}{\sigma_{r}}$, by normalizing the reward against the statistics of the group, where $\mu_{r}$ and $\sigma_{r}$ were the mean and SD of the rewards in that group. Outputs with above-average rewards received a positive advantage, thereby incentivizing the model to learn a superior generation policy. Finally, the parameters θ were updated by maximizing the following GRPO objective function:


$$\begin{array}{rl} J_{GRPO}(\theta ) & =E_{v∼P(V)}E_{\left\{o_{i}{\}}_{i=1}^{G}∼\pi_{\theta_{old}}(\cdot |v\right)}\\ \;[\frac{1}{G}\sum_{i=1}^{G}\min\;\left(r_{i}^{ratio}A_{i},\;clip\;\left(r_{i}^{ratio},1\pm \epsilon \right)A_{i}\right)\\ \;-\beta D_{KL}\;\left(\pi_{\theta }(\cdot |v)\;\|\;\pi_{ref}(\cdot |v)\right)] \end{array}$$

This objective function leveraged the reward-based advantage signal and used a clipped importance sampling ratio, $r_{i}^{\text{ratio}}$, to ensure training stability. Additionally, a Kullback-Leibler (KL) divergence regularization term, $D_{KL}(\pi_{\theta }\vee \pi_{\text{ref}})$, penalized significant deviations from the initial reference model $\pi_{\text{ref}}$ to prevent catastrophic forgetting. The model was then updated via back propagation, completing the training loop.

### SFT-Low-Rank Adaptation Baseline

To evaluate the efficacy of our proposed GRPO-based approach, we established a strong baseline using SFT, with the evaluation framework illustrated in Figure 3B. We applied this method to several VLMs, including Qwen2.5-VL-7B-Instruct, Llava-v1.6-Vicuna-7B, and the same Qwen2-VL-2B used in our GRPO experiments for a direct comparison. The SFT process adapted these pretrained models by training them on a dataset of curated (input, output) pairs to maximize the likelihood of generating the ground-truth output.

To enhance computational efficiency and mitigate the risk of catastrophic forgetting, we used low-rank adaptation (LoRA). Instead of fine-tuning the entire set of model parameters, LoRA injects trainable, low-rank matrices into the transformer layers of the VLM. During training, only these lightweight matrices were updated, while the original pretrained weights remained frozen. For a direct and fair comparison, the training data for SFT mirrored the format used in our RL setup. Each ground-truth output consisted of a complete reasoning trace enclosed in <think>...</think> tags followed by a definitive answer in <answer>...</answer> tags. The model was trained by minimizing the standard cross-entropy loss between its predicted token sequence and the ground-truth sequence. Specifically, for a given ground-truth sequence $Y=\left(y_{1},y_{2},\ldots ,y_{T}\right)$, the SFT loss was formulated as the negative log-likelihood of the target tokens:

Where θ represents the trainable model parameters, x is the input, and $P_{\theta }\left(y_{t}|x,y_{t}\right)$ is the probability of the model predicting the correct token $y_{t}$ given the input and the preceding ground-truth tokens $y_{t}$. This SFT-LoRA baseline represented a standard and powerful adaptation technique, providing a crucial benchmark to assess the unique advantages of applying RL to this medical reasoning task.

### Quantization Methodology

To evaluate the deployment feasibility of ECG-R1 in resource-constrained clinical settings, we implemented posttraining quantization to reduce computational precision (from BF16 to INT8 or INT4). This technique substantially lowered video RAM (VRAM) requirements and increased inference speed. Specifically, we used the bitsandbytes library for INT8 quantization and the activation-aware weight quantization algorithm for INT4 [33]. Activation-aware weight quantization was selected to preserve the salience of model weights critical for interpreting complex ECG waveforms while minimizing memory use. This approach ensured an optimal balance between diagnostic performance and high-speed inference on low-cost hardware.

## Results

### Training and Implementation Details

**Figure 4.**
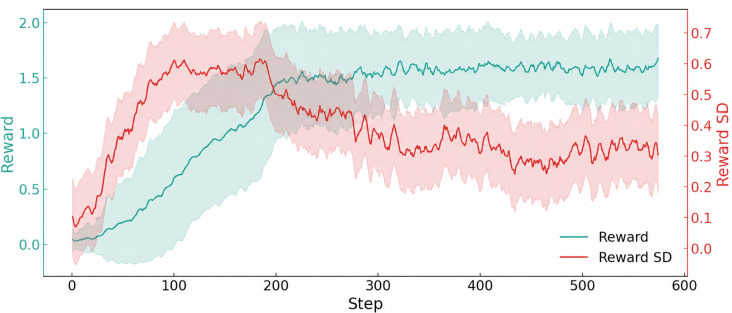
Group relative policy optimization reward and reward SD.

Our study used Qwen2-VL-2B as the base VLM. While this model was originally trained on a diverse corpus of web pages, open-source datasets, and synthetic data, we adapted it for the medical domain using the GRPO RL framework described in the methodology. Our implementation was built upon open-source VLM inference libraries [34-36]. The model underwent full fine-tuning for 594 steps on 4 NVIDIA A6000 graphics processing units (GPUs; 48 GB VRAM each). With a batch size of 1, the total training time was approximately 32 hours. For the GRPO algorithm, the number of G was set to 8. All other training and optimization hyperparameters were configured in accordance with the recommendations in [35]. The progression of the GRPO training process, demonstrating the continuous improvement in reward and the stabilization of the reward SD, is illustrated in Figure 4.

For our SFT baseline experiments, we used LoRA for the Qwen2-VL-2B, Qwen2.5-VL-7B-Instruct, and Llava-v1.6-Vicuna-7B models. To ensure a fair and consistent comparison, we used a unified LoRA configuration for all 3 models. Specifically, the LoRA rank was set to 16 with a scaling factor of 32. We applied LoRA to the query, key, value, and output projection layers within the transformer blocks, with a dropout rate of 0.01. These models were trained for 3 epochs using the AdamW optimizer with a learning rate of $2\times 10^{-4}$ and a batch size of 8. The training was also conducted on 4 NVIDIA A6000 GPUs.

Figure 4 tracks GRPO training for Qwen2-VL-2B over 594 steps. The rising reward (green) indicates continuous improvement in medical reasoning accuracy. The decreasing then stabilizing reward SD (red) reflects reduced performance variability as the policy converges, confirming stable and effective RL.

### Comparative Performance on TWI Reasoning

To comprehensively evaluate our proposed method, we compared its performance against several baselines on the ECG TWI reasoning task. The evaluation was conducted under 2 settings to assess generalizability: in-domain, where the model was trained and tested on data from the same hospital (eg, hospital A→hospital A), and out-of-domain, where the model was trained on data from one hospital and tested on data from another (eg, hospital A→hospital B). This cross-hospital validation served as a stringent test of the ability of the model to generalize to unseen data distributions.

The comprehensive clinical metrics (accuracy, sensitivity, specificity, positive predictive value, negative predictive value, and AUC-ROC) are summarized in Table 1 across 3 model categories: zero-shot/ViTs, SFT VLMs (baselines), and our GRPO VLM. As the results show, zero-shot models performed near random chance. While vision-only baselines (eg, ViT-L/14) established a functional 62.21% in-domain accuracy, they were consistently outperformed by multimodal SFT models, confirming that the synergistic integration of visual waveforms and clinical text was essential for diagnostic precision.

**Table 1.** Baseline characteristics of the study population stratified by data source.

Characteristic	In-domain dataset (hospital A; n=12,917)	Out-of-domain dataset (hospital B; n=1356)	Overall (n=14,273)
Demographics			
Age (years), mean (SD)	62.5 (10.4)	63.1 (11.2)	62.6 (10.5)
Male, n (%)	8396 (65)	854 (63)	9250 (64.8)
Patient source, n (%)			
Inpatient	9042 (70)	1017 (75)	10,059 (70.5)
Outpatient and emergency	3875 (30)	339 (25)	4214 (29.5)
Class distribution, n (%)			
Ischemic TWI^a^–positive	6187 (47.9)	651 (48)	6838 (47.9)
Nonischemic TWI–negative	6730 (52.1)	705 (52)	7435 (52.1)
Key comorbidities, n (%)			
Hypertension	7854 (60.8)	835 (61.6)	8689 (60.9)
Diabetes mellitus	3953 (30.6)	405 (29.9)	4358 (30.5)
Dyslipidemia	5800 (44.9)	612 (45.1)	6412 (44.9)
Prior myocardial infarction	1653 (12.8)	184 (13.6)	1837 (12.9)

a TWI: T-wave inversion.

Although SFT baselines demonstrated strong in-domain capabilities (eg, Llava-v1.6-Vicuna-7B achieved 69.40% accuracy and 73.41% AUC-ROC), they suffered notable performance drops in the out-of-domain setting (accuracy degrading to 62.33%), indicating overfitting to the source hospital’s specific artifacts. In stark contrast, our GRPO-based approach achieved a new state-of-the-art performance. It achieved the highest in-domain accuracy (75.21%) and AUC-ROC (84.18%), with an exceptional sensitivity of 82.55% that effectively minimized critical false negatives. While the in-domain specificity of ECG-R1 (68.43%) was marginally lower than certain SFT baselines such as Qwen2-VL-2B full-FT (72.58%), this represented a deliberate algorithmic trade-off; prioritizing sensitivity over specificity was an essential design feature for a frontline triage tool. Crucially, the GRPO model exhibited remarkable out-of-domain robustness, maintaining 72.93% accuracy and 81.56% AUC-ROC. This minimal cross-hospital degradation proved that optimizing for clinical reasoning via RL allowed the model to learn invariant, generalizable physiological features rather than overfitting to surface-level patterns.

**Figure 5.**
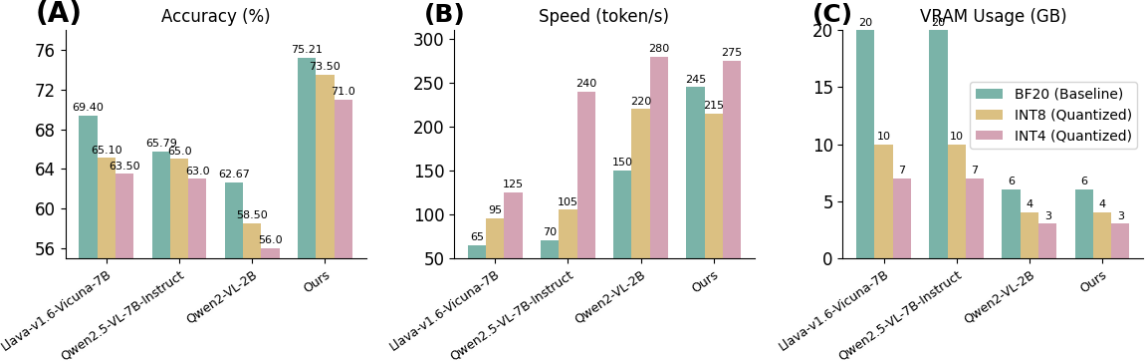
Comprehensive evaluation of vision-language model performance under quantization. The charts illustrate the trade-offs between (A) accuracy (%), (B) inference speed (token/s), and (C) video RAM (VRAM) use (GB) at different precision levels (BF16, INT8, and INT4).

As detailed in our methodology, all inference benchmarks—including inference speed and VRAM consumption (GB) visualized in Figure 5—were measured on a single NVIDIA GeForce RTX 4090 GPU with 24 GB of memory. The trade-offs between accuracy, speed, and VRAM use across different models and quantization levels are visualized in Figure 5. Compared with the larger 7B models, ECG-R1 maintained the highest accuracy across all quantization levels. This demonstrated that ECG-R1 can achieve high-speed inference on low-cost hardware, striking an optimal balance between diagnostic performance and resource consumption.

**Figure 6.**
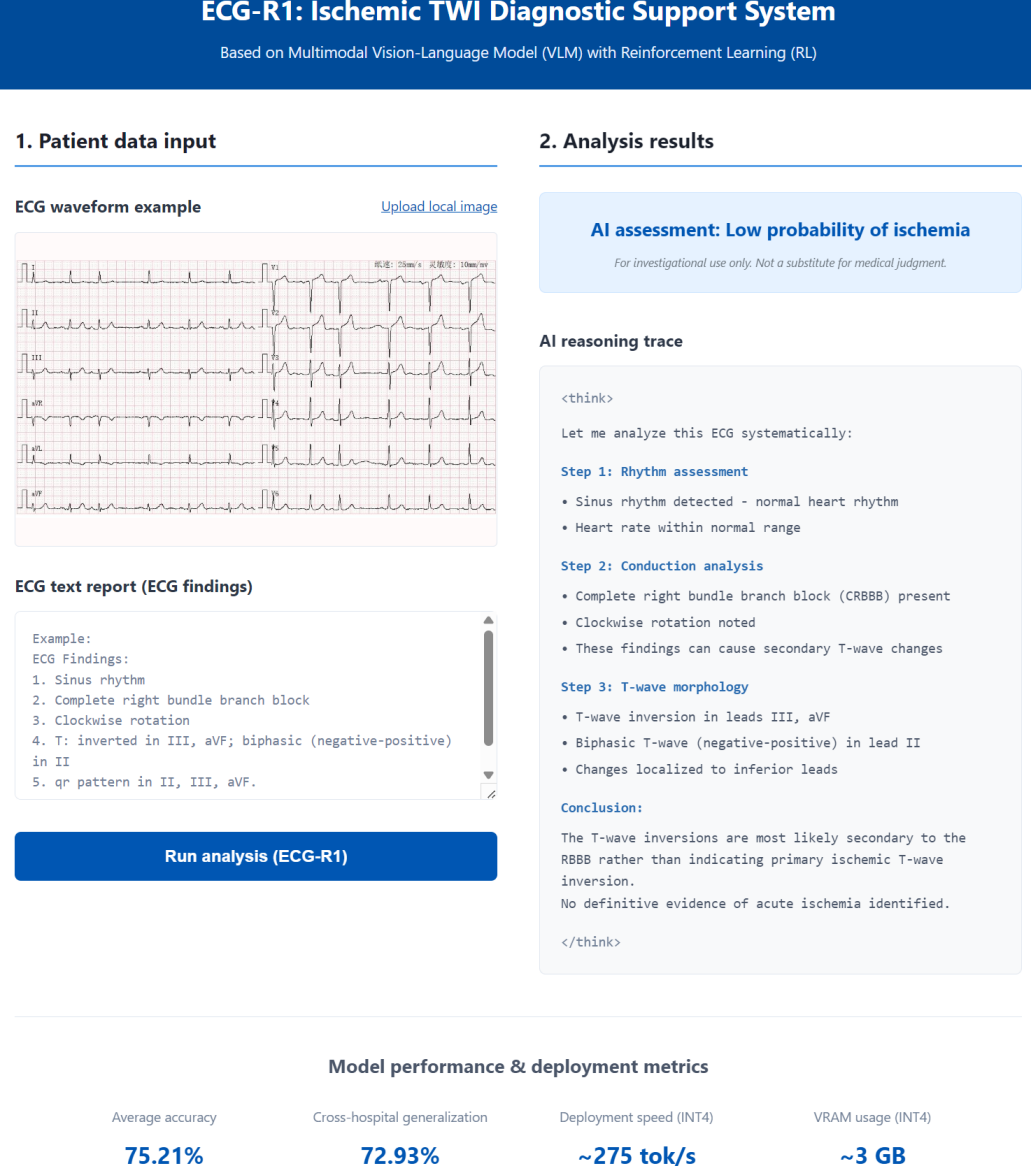
Demonstration of the ECG-R1 clinical decision support interface. To align with responsible artificial intelligence (AI) practices and prevent premature cognitive closure, the system uses probabilistic language. The underlying continuous probability score is mapped to risk-stratified categories: P(yes)≥0.80 as “high probability,” 0.20‐0.79 as “intermediate,” and <0.20 as “low probability.” The output panel displays a risk-stratified “AI Assessment,” accompanied by the interpretable reasoning trace (the <think>... block). This transparent design ensures the attending physician can independently verify the AI’s logic and retain ultimate diagnostic authority. ECG: electrocardiogram.

Furthermore, our framework directly addressed the critical need for transparency and safety in medical AI. By integrating multimodal inputs with a reward structure that mandates an explicit reasoning trace, the model’s decision-making process became highly interpretable. As illustrated in the clinical decision support interface (Figure 6), the system was intentionally designed to output probabilistic assessments rather than definitive diagnoses. To achieve this, while the model was prompted to generate a definitive “yes” or “no” answer token, the inference engine extracted the underlying softmax confidence score (log probabilities) of this final generated token. These mathematically derived, continuous probability scores were then mapped to risk-stratified textual categories (eg, “low probability of ischemia”) presented in the user interface. This structural choice was vital to mitigate the risk of premature cognitive closure, ensuring the tool acted strictly as an assistive triage flag that complemented, rather than replaced, human clinical judgment.

### Qualitative Evaluation of Reasoning

**Figure 7.**
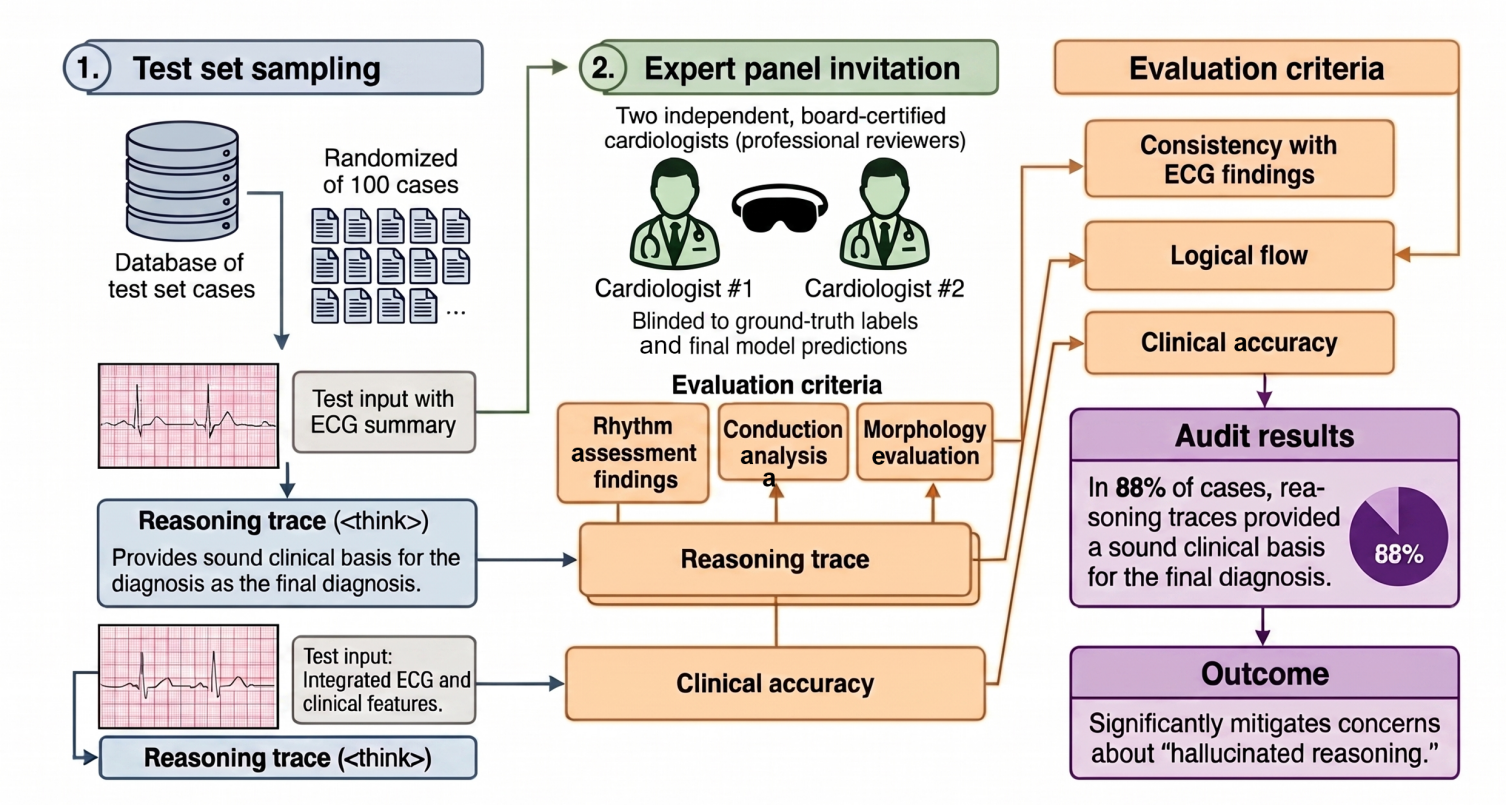
Schematic illustration of the blinded qualitative audit workflow for evaluating generated reasoning traces. ECG: electrocardiogram.

As illustrated in Figure 7, to assess the clinical validity of the reasoning, we conducted a blinded qualitative audit on 100 randomly sampled test cases. Two independent, board-certified cardiologists reviewed the generated reasoning traces (**<**think**>** blocks) based on 3 specific criteria: consistency with ECG findings, logical flow, and clinical accuracy. The reviewers remained strictly blinded to ground-truth labels and predictions to ensure objectivity. Audit results demonstrated that in 88% of cases, the traces provided a sound clinical basis for the diagnosis, significantly mitigating concerns about “hallucinated reasoning” and confirming that the model’s decision-making was grounded in valid clinical evidence.

### Ablation Results

To verify that ECG-R1 did not rely on linguistic shortcuts, we compared it against image-only models. As shown in Table 2, the ViT-B/16 and ViT-L/14 models achieved in-domain accuracies of 60.18% and 62.21%, respectively. The superior performance of ECG-R1 (75.21%) over these image-only baselines demonstrated that the RL paradigm successfully leveraged the complementary information between visual waveforms and objective clinical text to achieve higher diagnostic precision.

**Table 2.** Performance comparison of the proposed group relative policy optimization (GRPO) vision-language model (VLM) against zero-shot, vision-only, and supervised fine-tuning (SFT) baselines on in-domain and out-of-domain datasets.^a^

Method	Test domain	Accuracy (%; 95% CI)	Sensitivity (%; 95% CI)	Specificity (%; 95% CI)	PPV^b^ (%)	NPV^c^ (%)	AUC-ROC^d^ (95% CI)
Zero-shot							
Llava-v1.6-Vicuna-7B	In-domain	48.55 (46.9‐50.1)^e^	44.44 (42.1‐46.7)	52.34 (50.1‐54.5)	46.26	50.51	50.12 (48.5‐51.7)
Qwen2-VL-2B	In-domain	31.34 (29.9‐32.8)^e^	15.25 (13.6‐16.9)	46.19 (43.9‐48.5)	20.74	37.12	37.85 (36.3‐39.4)
Qwen2.5-VL-7B	In-domain	44.10 (42.5‐45.7)^e^	48.35 (46.1‐50.6)^e^	40.18 (38.0‐42.4)^e^	42.73	45.73	46.54 (44.9‐48.1)^e^
Vision transformer							
ViT-B/16 (vision only)	In-domain	60.18 (58.6‐61.7)^e^	56.25 (53.9‐58.5)^e^	63.80 (61.7‐65.9)^e^	59.10	61.20	63.85 (62.3‐65.3)^e^
ViT-B/16 (vision only)	Out-of-domain	54.94 (52.3‐57.6)^e^	51.40 (47.6‐55.2)^e^	58.20 (54.5‐61.9)^e^	53.40	56.30	58.20 (55.5‐60.9)^e^
ViT-L/14 (vision only)	In-domain	62.21 (60.6‐63.7)^e^	58.75 (56.4‐61.0)^e^	65.40 (63.3‐67.4)^f^	61.35	63.15	65.60 (64.1‐67.1)^e^
ViT-L/14 (vision only)	Out-of-domain	57.25 (54.6‐59.9)^e^	54.10 (50.3‐57.9)^e^	60.15 (56.4‐63.9)^f^	55.80	58.60	60.40 (57.7‐63.1)^e^
SFT VLM (baseline)							
Llava-v1.6-Vicuna-7B (LoRA^g^)	In-domain	69.40 (67.9‐70.8)^e^	66.85 (64.6‐69.0)^e^	71.75 (69.7‐73.7)^h^	68.60	70.10	73.41 (72.0‐74.8)^e^
Llava-v1.6-Vicuna-7B (LoRA)	Out-of-domain	62.33 (59.7‐64.9)^e^	59.43 (55.6‐63.2)^e^	65.01 (61.4‐68.5)	61.06	63.45	66.82 (64.3‐69.3)^e^
Qwen2-VL-2B (full-FT)	In-domain	68.52 (67.0‐69.9)^e^	64.12 (61.9‐66.3)^e^	72.58 (70.6‐74.5)^f^	68.34	68.67	72.15 (70.7‐73.5)^e^
Qwen2-VL-2B (full-FT)	Out-of-domain	61.45 (58.8‐64.0)^e^	58.20 (54.4‐61.9)^e^	64.45 (60.8‐68.0)	60.18	62.55	65.40 (62.8‐67.9)^e^
Qwen2-VL-2B (LoRA)	In-domain	62.67 (61.2‐64.2)^e^	55.42 (53.2‐57.6)^e^	69.36 (67.4‐71.3)	62.54	62.76	68.12 (66.6‐69.6)^e^
Qwen2-VL-2B (LoRA)	Out-of-domain	55.81 (53.2‐58.4)^e^	48.75 (45.0‐52.5)^e^	62.33 (58.8‐65.8)	54.43	56.85	60.54 (57.9‐63.1)^e^
Qwen2.5-VL-7B (LoRA)	In-domain	68.12 (66.6‐69.5)^e^	65.34 (63.1‐67.5)^e^	70.69 (68.7‐72.6)	67.30	68.84	71.25 (69.8‐72.6)^e^
Qwen2.5-VL-7B (LoRA)	Out-of-domain	63.45 (60.8‐66.0)^e^	60.18 (56.4‐63.9)^e^	66.47 (62.9‐69.9)	62.36	64.39	67.93 (65.4‐70.4)^e^
GRPO VLM (our study)							
Ours (Qwen2-VL-GRPO)	In-domain	75.21 (73.8‐76.6)	82.55 (80.8‐84.3)	68.43 (66.4‐70.4)	70.71	80.95	84.18 (83.0‐85.3)
Ours (Qwen2-VL-GRPO)	Out-of-domain	72.93 (70.5‐75.3)	79.80 (76.7‐82.9)	66.59 (63.1‐70.0)	68.80	78.12	81.56 (79.4‐83.6)

a The proposed GRPO VLM (ours) serves as the reference group for statistical comparisons. Statistical significance is determined using the McNemar test for accuracy, sensitivity, and specificity and the DeLong test for AUC-ROC.

b PPV: positive predictive value.

c NPV: negative predictive value.

d AUC-ROC: area under the receiver operating characteristic curve.

e *P*<.001.

f *P*<.01.

g LoRA: low-rank adaptation.

h *P*<.05.

## Discussion

### Principal Findings

This study introduced ECG-R1, a multimodal RL model designed to address the significant clinical challenge of differentiating ischemic from nonischemic TWI. It confronted the limitations of existing deep learning models, namely, their single-modality reliance, “black box” nature, and poor generalization. Our results provided strong evidence for the superiority of the RL-based approach over SFT baselines. Critically, while SFT models exhibited poor performance in out-of-domain (cross-hospital) evaluations, likely due to “rote memorization,” our GRPO-trained model (75.21% in-domain accuracy) demonstrated substantially better generalization. This suggested that RL incentivized the model to learn more robust pathological patterns. Furthermore, the strategic selection of a 2-billion parameter model achieved a pragmatic balance between high performance and clinical deployment feasibility, rendering it suitable for resource-constrained hospital environments. Finally, the framework successfully addressed the transparency challenge in medical AI by mandating explicit reasoning traces via an effective 2-component reward function assessing both format and accuracy. Acting as an automated triage tool to flag high-risk ischemic patterns, the model generates independently verifiable reasoning traces to mitigate automation bias. This structural transparency ensures that the attending physician consistently retains ultimate diagnostic authority and legal liability.

Real-world clinical text inherently contains noise, including abbreviations (Table S1 in Multimedia Appendix 1), typos, and missing information. Our multimodal VLM handled these issues through 2 key mechanisms. First, the base model’s extensive pretraining provided robustness against linguistic irregularities and clinical shorthand. Second, the multimodal architecture ensured diagnostic redundancy; when text was fragmented or absent entirely, the model dynamically shifted its attention to extract compensatory features directly from the ECG waveform, maintaining stable diagnostic performance.

### Limitations

While our findings demonstrated the significant potential of the ECG-R1 framework, several critical limitations must be addressed before clinical deployment. Although our cross-hospital validation established a robust baseline for generalizability, the dataset was sourced from only 2 institutions. Because large VLMs are pretrained on vast, uncurated internet corpora, they may inherently harbor demographic or socioeconomic biases. Consequently, future research must validate the model across larger, multicenter cohorts and conduct comprehensive fairness audits evaluating performance across diverse racial, ethnic, and age groups to prevent the perpetuation of health care disparities. Additionally, addressing the current error rate of approximately 24.79% was paramount. In cardiology, the clinical cost of errors is highly asymmetric; false negatives can lead to severe, life-threatening outcomes. Future iterations must therefore be strictly calibrated to prioritize sensitivity, ensuring the system operated as a highly conservative safety net.

A fundamental algorithmic constraint of the current framework lay in its reward mechanism. The rule-based system functioned effectively as an outcome-supervised reward model, optimizing primarily for structural formatting and the final diagnostic label. This sparse reward signal left the model vulnerable to “reward hacking,” a phenomenon where the model generated plausible but medically flawed intermediate logic to arrive at the correct final answer. In our current framework, this risk was implicitly mitigated by the KL divergence regularization term within the GRPO objective. Because the policy was anchored to an SFT reference model trained on expert-verified reasoning traces, the KL penalty effectively prevented severe semantic degradation or spurious keyword repetition. Overcoming this bottleneck required a transition to process-supervised reward models (PRMs). Implementing PRMs would provide a dense reward signal capable of explicitly evaluating and guaranteeing the logical validity of each step within the clinical reasoning chain, thereby securing the integrity of the model’s interpretability. Because developing PRMs was highly resource intensive, demanding expert step-by-step annotation, practical interim strategies included rigorous post hoc clinical audits (as in our study) or “AI-as-a-Judge” pipelines to automatically score reasoning steps.

Furthermore, using CAG as the ground truth introduced a verification bias, skewing the dataset toward patients with a higher pretest probability of ischemia. Consequently, our reported metrics primarily reflected a higher-risk subpopulation, as benign or asymptomatic TWI cases were underrepresented.

Finally, the retrospective design of this study implied that the true utility of the system within a live clinical workflow remained unproven despite its high offline diagnostic precision. The essential next step is the rigorous evaluation of ECG-R1 within prospective, randomized clinical trials to measure its tangible impact on physician decision-making and patient outcomes. Furthermore, addressing the complex ethical and liability concerns of AI deployment dictates a strict “human-in-the-loop” paradigm. To ensure clinical safety, the system must function exclusively as an assistive decision support tool—such as an automated triage flag—guaranteeing that the attending physician retains ultimate diagnostic authority and legal responsibility.

### Conclusions

In conclusion, our study demonstrated that applying RL to multimodal VLMs is a highly effective strategy for complex medical reasoning tasks. The ECG-R1 framework enhanced diagnostic accuracy, improved generalization, and promoted transparency, offering a powerful tool to augment clinical expertise and mitigate the risks of misdiagnosis in cardiology.

## Supplementary material

10.2196/87227 Multimedia Appendix 1 Representative deidentified examples of the clinical text paired with electrocardiogram images.

## Abbreviations

AI: artificial intelligence
AUC-ROC: area under the receiver operating characteristic curve
CAG: coronary angiography
ECG: electrocardiogram
GPU: graphics processing unit
GRPO: group relative policy optimization
KL: Kullback-Leibler
LoRA: low-rank adaptation
PRM: process-supervised reward model
RL: reinforcement learning
SFT: supervised fine-tuning
TWI: T-wave inversion
VLM: vision-language model
VRAM: video RAM
