# Supplementary material for: Differentiating Ischemic From Nonischemic T-Wave Inversion Using a Multimodal Vision-Language Model With Reinforcement Learning (ECG-R1): Development and Validation Study
Source: JMIR Med Inform. 2026 Jun 19;14:e87227. doi: 10.2196/87227 (PMC13281817; doi:10.2196/87227)
Supplement: Multimedia Appendix 1 [file medinform-v14-e87227-s001.docx]

Supplementary Table S1

**Representative de-identified examples of the clinical text paired with ECG images**

**Note.** The examples below are representative de-identified examples included to illustrate the textual modality used by the VLM. They are provided for transparency and are not intended to reproduce verbatim full patient records.

| **Example** | **Representative clinical text example** | **What the example illustrates** |
| --- | --- | --- |
| **Example 1** | **Cardiologist impression:** Sinus rhythm. ST segment horizontally depressed 0.5-1.5mm in I, aVL, V5, V6. T-wave biphasic in I, aVL, V6, notched in V4, and inverted in V5.  **Brief history/context:** 68-year-old male. Presenting symptom: chest tightness. | Demonstrates a properly redacted input where any conclusive diagnostic text (e.g., "concerning for ischemia") has been systematically scrubbed. The model is forced to rely purely on basic symptoms and raw morphological descriptions. |
| **Example 2** | **Cardiologist impression:** Sinus rhythm with premature atrial complexes. Complete Right Bundle Branch Block (CRBBB). T-wave inverted in III, biphasic in aVF and V4, notched in V5.  **Brief history/context:** 73-year-old male. Presenting symptom: chest tightness. | Illustrates a typical input for secondary non-ischemic TWI. Objective conduction abnormalities (CRBBB) are preserved as essential ECG findings, while ensuring no explicit diagnostic labels are included to prevent modality leakage. |

**Abbreviations.** CRBBB = complete right bundle branch block; TWI = T-wave inversion.

**Integration note.** In the multimodal pipeline, the rigorously redacted case-specific clinical text was concatenated with the fixed instruction prompt and paired with the corresponding ECG image. This explicit scrubbing ensures the model learns cross-modal features without risk of text-modality leakage from diagnostic labels.
